# Qualitative and Quantitative Analysis on Flavonoid Distribution in Different Floral Parts of 42 *Hemerocallis* Accessions

**DOI:** 10.3389/fpls.2021.670506

**Published:** 2021-05-07

**Authors:** Sen Li, Huliang Cui, Jinyao Wang, Feifan Hou, Xiong Xiong, Xiuping Kang, Guoming Xing

**Affiliations:** ^1^College of Horticulture, Shanxi Agricultural University, Jinzhong, China; ^2^Collaborative Innovation Center for Improving Quality and Increase of Protected Vegetables in Shanxi Province, Jinzhong, China; ^3^Datong Daylily Industial Development Research Institute, Datong, China

**Keywords:** *Hemerocallis*, flavonoids, floral organ, HPLC, qualitative, quantitative

## Abstract

The *Hemerocallis* accessions is widely consumed as nutritious vegetable and traditional medicine in eastern Asia and used as an ornamental flower worldwide. Compared with most other horticultural products, its flower is richer in polyphenols, flavonoids, carotenoids, and anthocyanins. Therefore, the flower has strong antioxidant activity that inhibits cancer cell proliferation, which could used for health and pharmaceutical purposes. The flavonoids composition and distribution in the flowers, and the content varied between different accssions is still unclear. In this context, eight flavonols, two flavones, and two anthocyanins were determined in *Hemerocallis* flower by high-performance liquid chromatography (HPLC) coupled with photodiode array and mass spectrometric detectors. Rutin was the most abundant flavonols and cyanidin 3,5-glucoside and cyanidin 3-rutinoside were the major anthocyanins in *Hemerocallis* tepals, resulting in flower petal coloration, and their content in the petal was higher than that of the sepal. Hierarchical cluster analysis grouped the 42 accessions into four groups, and they were significantly different (*p* < 0.05) from each other in the ten significant compounds by One-way ANOVA. Overall, the qualitative and quantitative analysis of flavonoid constituents in six floral parts of 42 *Hemerocallis* accessions were elucidated, which could be helpful for the food and pharmaceutical industries, and lay the foundation for the *Hemerocallis* flower color research.

## Highlights

- First report on systematic identification and quantification of flavonoid in *Hemerocallis* floral organs.
- Eight flavonols, two flavones, and two anthocyanins in *Hemerocallis* flower, and Rutin was the dominant flavonol.
- Our data are helpful for the *Hemerocallis* flower used for the food and pharmaceutical industries.

## Introduction

*Hemerocallis* spp. are ornamental herbaceous perennials with more than 83,000 modern cultivars in the world (Wang and Gao, 2014). These *Hemerocallis* accessions have a cultivation history of more than 2,000 years in China, where is the distribution center of *Hemerocallis* in the world. *Hemerocallis citrina* is a traditional vegetable. According to our preliminary statistics, its cultivated area in China exceeds 73,000 hectares, with an annual production of 80,000 tons. Therefore, there is considerably economic value in *H. citrina.* Besides, *Hemerocallis* is mainly used for landscape beautification and is also an important cut flower material. These plants grow well in different soil types and can bloom normally under either full sun or light shade. Hence, from the cold temperate zone to the tropics can see a large number of applications of *Hemerocallis*.

*Hemerocallis* have been widely consumed as nutritious food and traditional medicine in eastern Asia (Rodriguez-Enriquez and Grant-Downton, 2013). The edible part of *Hemerocallis* is the flower bud growing on top of the floral axis and typically has more than 20 flowers per scape. Compared with most other vegetables, its flower is richer in polyphenols (Lin et al., 2011), flavonoids (Fu and Mao, 2006; Lin et al., 2011), carotenoids (Tai and Chen, 2000; Hsu et al., 2011), and anthocyanins (Tai and Chen, 2000; Deng et al., 2003; Fernandes et al., 2017). The flowers also have strong antioxidant activity that inhibits cancer cell proliferation (Cichewicz et al., 2004; Kao et al., 2015), hence it is used for health and pharmaceutical purposes. This perennial herb is projected to have substantial market prospects in the future (Wiseman et al., 1996; Zloch, 1996; Cos et al., 2004; Manach et al., 2004).

The variation of *Hemerocallis* flower color is abundant (Li et al., 2016), and the color of petals and sepals may also be different. Therefore, *Hemerocallis* is an ideal material in flower color research. The flower color is the result of metabolite accumulation in the vacuoles of flower epidermal cells (Wang et al., 2018). Flavonoid metabolism pathways play important roles in modulating plant color. The differences in the presence, quantity or type of flavonoid pigments is one of the main reasons for the yellow flower color (Deng et al., 2013). However, the differential accumulation of flavonols, flavones, and anthocyanins in different *Hemerocallis* germplasm remain unclear. In addition, there are few reports on the qualitative and quantitative analysis of the secondary metabolites especially flavonoids in *Hemerocallis* flower parts, which greatly slows down the process of its flower color breeding and restricts its economic and industrial development.

In this study, we collected 42 *Hemerocallis* accessions with different colors and origins. Our aim is to (1) assess the flavonoid composition and content in different floral parts of *Hemerocallis* and (2) evaluate variations in the flavonoid contents of the different accessions. These research results provide a theoretical basis for analyzing the accumulation of *Hemerocallis* flavonoids and a scientific reference for exploring its edible value by food and pharmaceutical industries.

## Materials and Methods

### Plant Materials

**FIGURE 1:**
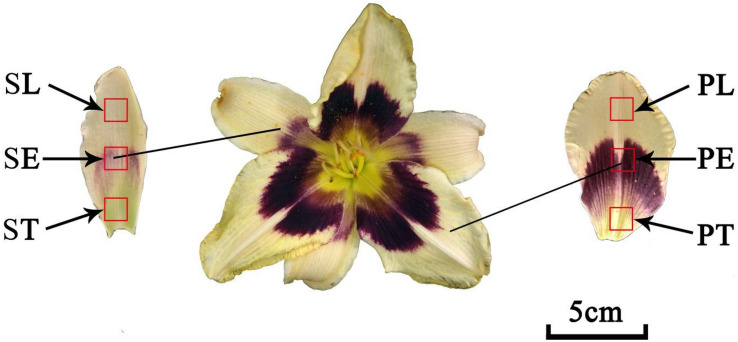
The flower organs of daylily. ST, sepal throat; SE, sepal eye; SL, sepal limb; PT, petal throat; PE, petal eye; PL, petal limb.

A total of 42 accessions were used in this study. These accessions were from different geographic regions around the world including commercial accessions and landraces. The details of these 42 accessions were presented in Supplementary Table 1 and Table 1. The sepals and petals were collected from each accession and divided into six parts (Figure 1), sepal throat (ST), sepal eye (SE), sepal limb (SL), petal throat (PT), petal eye (PE), and petal limb (PL), according to a previously described method (Cui et al., 2019). Subsequently, each part was placed in liquid nitrogen immediately after detaching, then preserved at −80°C until the flavonoids extraction.

**TABLE 1:** The 42 *Hemerocallis* accessions used in this study.

Groups	Floral color	Num	Accessions
Night Lilies	Yellow	15	‘Datonghuanghua’ (1), ‘Yeshenghuanghua’ (2), ‘Qiaotouhuanghua’ (3), ‘Dongzhuanghuanghua’ (4), ‘Shezhuanghuanghua’ (5), ‘Dalihuanghua’ (6), ‘Xianhuanghua’ (7), *H. minor* (8), ‘Malinhuanghua’ (9), ‘Huohuanghua’ (10), ‘Yanchihuanghua’ (11), ‘Chazihua’ (12), ‘Qiezi’ (13), ‘Panlonghua’ (14), *H. citrine* (15)
Daylilies	Yellow	6	‘Double Cutie’ (16), *H. thunbergii* (17), ‘Nakai’ (18), ‘Little Bee’ (19), ‘Beijing-7’ (20), ‘Beijing-9’ (21)
	Pink	5	‘Canadian Border Patrol 2’ (22), ‘Always Afternoon’ (23), ‘Lullaby Baby’ (24), ‘Canadian Border Patrol’ (25), ‘Green Mystique’ (26)
	Orange	5	*H. fulva* var. *kwanso* var. *reasata* (27), ‘Bonanza’ (28), ‘Childrens Festival’ (29), ‘Dahuaxuancao’ (30), *H. altissima* (31)
	Red	6	‘Baltimore Oriole’ (32), *H. aurantiaca* (33), ‘Red Cloud’ (34), ‘Little Wine Cup’ (35), ‘Austria Ruby’ (36), ‘Wenxixuancao’ (37)
	Purple	4	‘Purple Gems’ (38), ‘Blazing sun’ (39), ‘Blue Sheen’ (40), ‘Elegant Greeting’ (41)
	Bicolor	1	‘Frans Hals’ (42)

*Numbers in parentheses represented accession no.*

### Reagents and Chemicals

High-performance liquid chromatography grade methanol, formic acid, trifluoroacetic acid (TFA), and acetonitrile were purchased from Fisher Scientific (Fair Lawn, NJ). Quercetin 3-glucoside (Qu3g), myricetin, kaempferol 3-glucorhamnoside, apigenin, and two cyanidin derivatives standards, cyanidin 3-glucoside (Cy3g), and cyanidin 3-rutinoside (Cy3r) were purchased from Sigma-Aldrich (St. Louis, MO). Kaempferol (Km), quercetin (Qu), and rutin (Rt) were purchased from Solarbio (Solarbio, China). Ultrapure water from PureLab Ultra Water System (ELGA LabWater, United Kingdom) was used in this experiment. All other reagents used were of analytical grade.

### Extraction and HPLC Analysis of Flavonoids and Anthocyanins

Samples (0.1 g fresh weight, FW) were fully ground in liquid nitrogen, extracted in 2 mL solvent mixture (methanol/water/formic acid/TFA, 70:27:2:1, v/v/v/v), and allowed to settle for 24 h without light. The extracts were then centrifuged (12,000 rpm, 20 min), and the supernatant was filtered (0.22 μm) into vials. The HPLC analyses were carried out on a Thermo Fisher HPLC system connected with a 996 photodiode array detector (UltiMate 3000, ThermoFisher, United States), which was set in the range of 190–600 nm. Data collection and processing was accomplished by the Chameleon software version 2.0. The chromatographic separation was performed on a Venusil ASB C18 column (Agela Technologies, China) with 4.6 mm × 250 mm, 5 μm. The mobile phases comprised 2% aqueous formic acid (A) and acetonitrile (B). The gradients were programed as follows: 0 min, 8% B; 3 min, 8% B; 23 min, 20% B; 33 min, 40% B; 43 min, 40% B; 45 min, 8% B. The column temperature, injection volume, and flow rate were set at 35°C, 10 μL, and 0.8 mL/min, respectively. The flavonoids and anthocyanin chromatograms were extracted at 350 nm and 520 nm. All samples were extracted in triplicate.

### LC–MS Analysis of Flavonoids and Anthocyanins

Liquid chromatography–mass spectrometry (LC–MS) analysis was performed on the HPLC instrument described above, interfaced with a microOTOF Q quadrupole time-of-flight mass spectrometer (Thermo Fisher, United States) connected to either electrospray ionization (ESI) or an atmospheric pressure chemical ionization source. The HPLC analysis conditions were similar to those described above. The mass signal range was *m/z* 50–1,100. The ionization of flavonoids was achieved with an ESI source in both positive and negative modes, and the parameters were set as follows: capillary voltage, 3,500 V; endplate offset, 500 V; drying gas (nitrogen) flow, 8.0 L/min; drying gas temperature, 180°(C; collision rf, 200 Vpp; nebulizer pressure, 0.8 bar; prepulse storage, 8.0 (*s*; transfer time, 80.0 μ*s*; and collision energy, 10.0 eV.

### Quantitation of Flavonoids and Anthocyanins

The quantitation was conducted by external calibration of the corresponding standards from the areas of the chromatographic peaks at 350 nm for flavonoids and 520 nm for anthocyanins. All standards were dissolved in methanol. The following equations were used: cyanidin-3-glucoside (*y* = 274.1046 *x* + 0.1328, *R*^2^ = 0.99987); quercetin (*y* = 392.9441 *x* + 0.5815, *R*^2^ = 0.99865); quercetin 3-glucoside (*y* = 339.3973 *x* − 0.3364, *R*^2^ = 0.99997); rutin (*y* = 214.2924 *x* − 0.1913, *R*^2^ = 0.98327); apigenin (*y* = 516.2105 *x* + 0.0001, *R*^2^ = 0.99666); myricitrin (*y* = 1040.74 *x* − 2.1097, *R*^2^ = 0.99798). The content of compounds that did not have corresponding standards was calculated from the most suitable standard calibration curve.

### Statistical Analysis

The mean value of each sample was obtained from three replications and used for further analysis. The statistical analysis was conducted by the R (*x*64 3.5.1) software. The variations in the contents of ten major flavonoids among different floral parts were determined by the Mann–Whitney *U* test at *p* < 0.001. The hierarchical cluster analysis was conducted by the Ward D method, and the flavonoids difference between clusters were determined by one-way ANOVA at *p* < 0.05.

## Results and Discussion

### Identification of Flavonoids

**FIGURE 2:**
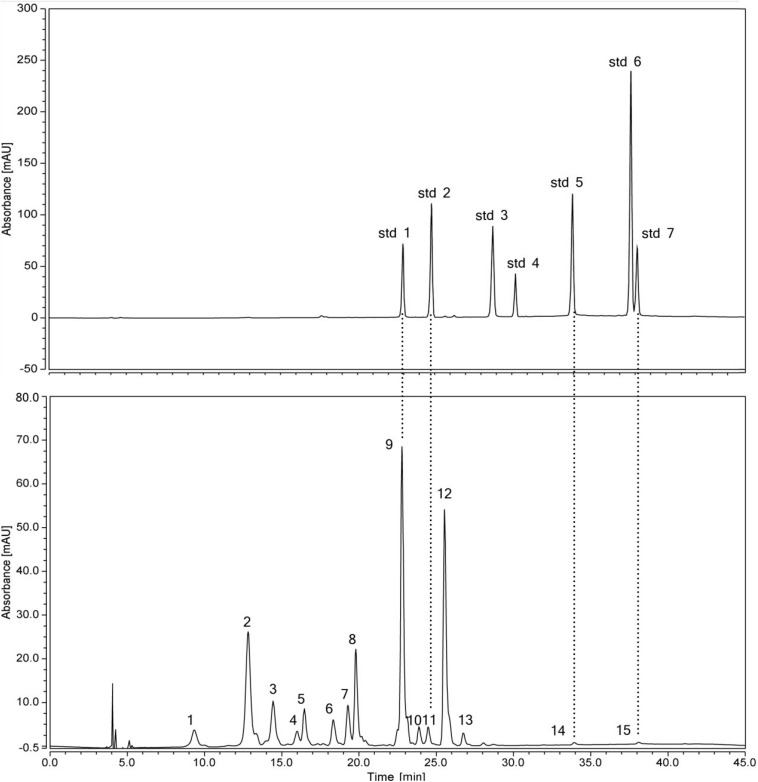
HPLC chromatogram of a mix of standard flavonoids and flavonoids extractd from the petal eye of ‘Little Bumble Bee’ (detection at 350 nm). Std1, rutin; std2, quercetin 3-glucoside; std3, myricetin; std4, kaempferol 3-glucorhamnoside; std5, quercetin; std6, apigenin; std7, kaempferol. Peaks numbers were shown in Table 2.

**FIGURE 3:**
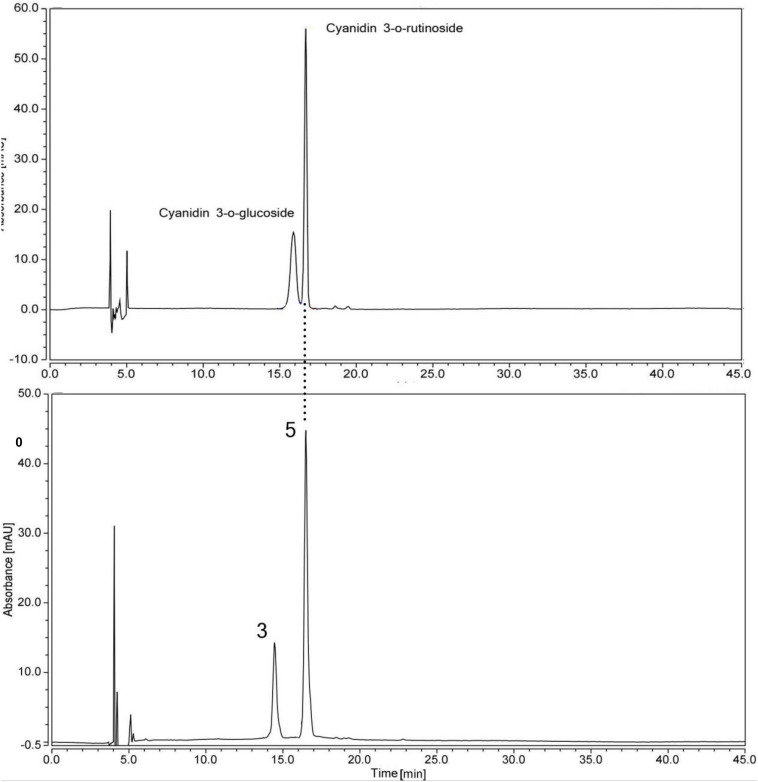
HPLC chromatogram of a mix of standard anthocyanins and flavonoids extracted from the petal eye of ‘Little Bumble Bee’ (detection at 520 nm). Peak numbers were the same as shown in Table 2.

The flavonoids were identified according to HPLC retention times, UV λ_max_ spectrum, and MS data (in both NI and PI modes), as well as previous reports (Markham, 1989). In total, 15 flavonoids were detected in the 42 accessions. The HPLC-DAD and HPLC-ESI(±)-MS^2^ analyses results, such as molecular ion, aglycone ion, and main fragments of MS^2^, are summarized in Table 2. In this study, eight flavonols, two flavones, and two anthocyanins were identified from the flowers of *Hemerocallis*, and three compositions were unknown. The PE of *Hemerocallis* ‘Little Bumble Bee’ was of these 15 compositions (Figures 2, 3).

**TABLE 2:** Chromatographic, spectroscopic, and mass spectrometric features of flavonoids detected in this study.

Peaks	Time (min)	UV (nm)	Compounds	[M–H]+	[Y0]+	[M–H]−	[Y0]−	References
1	9.723	324.77	U1	663.01	479/317.01			
2	13.07	309.62	U2			609.11	300.03	
3	14.6	344.48,524.41	Cy3g5g	611.09	287.1			Zhang et al., 2014
4	16.04	326.99,365.95	Qu3ar			433.08	271/255.04	Sun et al., 2018; Chen et al., 2010
5	16.7	326.93,516.64	Cy3r	595.16	433.11/286.8			Std
6	18.39	306.02	U3	595.16	287.05			
7	19.35	311.18	Is3r			623.09	315.02	Deng et al., 2013
8	19.99	363.34	Qu7g			463.09	271	Pop et al., 2013; Sarangowa et al., 2014
9	22.95	353.94	Rt	609.1	301.04			Std
10	23.65	333.69	Lt7g	449.01	287/149.03			Sarangowa et al., 2014
11	24.07	345	Qu3g	463.09	271.09			Std
12	25.67	347.15	Km3g	449.01	287.04			Li et al., 2009; Sarangowa et al., 2014
13	26.9	345.35	Ap7g	433	271	431	268	Mitchell et al., 1998; Bączek et al., 2019
14	33.94	366.42	Qu	303.04	229.05			Std
15	38.74	340.47	Km			285.05	229.05	Std

*U1, indicates unknown 1; U2, indicates unknown 2; Cy3g5g, indicates cyanidin 3,5-glucoside; Qu3ar, indicates quercetin 3-arobinoside; Cy3r, indicates cyandin 3-rutinoside; U3, indicates unknown 3; Is3r, indicates isorhamnetin -3-rutinoside; Qu7g, indicates quercetin 7-glucoside; Rt, indicates rutin; Lt7g, indicates luteolin 7-glucoside; Qu3g, indicates quercetin 3-glucoside; Km3g, indicates kaempferol 3-glucoside; Ap7g, indicates apigenin 7-glucoside; Qu, indicates quercetin; and Km, indicates kaempferol.*

Thirteen of the fifteen separated peaks showed λ_max_ at 350 nm, indicating they were flavonols and flavones. According to four standard retention times (Figure 2) and MS data, the peaks 9, 11, 14, and 15 were speculated as rutin (Rt), quercetin 3-glucoside (Qu3g), quercetin (Qu), and kaempferol (Km), respectively. Rt, Qu, and Km were previously published in the alabastrum of *H. fulva* (Lin et al., 2011; Chen et al., 2012; Kao et al., 2015). Also, Qu3g is found in *H. fulva* fresh leaves (Yanjun et al., 2004) and ‘Stella de Oro’ flowers (Cichewicz and Nair, 2002). Leaves of different amaranth species also have abundant Rt, Qu, Qu3g, and Km (Sarker and Oba, 2018, 2019, 2020c,d).

In methanol, kaempferol exhibits λ_max_ spectrum at approximately 266 nm (band II) and 367 nm (band I), while the λ_max_ of quercetin was approximately 255 and 370 nm (Markham, 1989). Furthermore, the glycosylation of the 3-hydroxyls could cause a hypsochromic shift of band I by about 12–17 nm, whereas the 7-hydroxyls would not change the λ_max_ (Singh et al., 2010). Based on these principles, peak 8 was indicated as quercetin 7-glucoside (Qu7g) with λ_max_ spectrum (band I) at 363.3 nm, MS data 463.09 ([M–H]^+^), and fragment *m/z* 271 ([Y0]^+^), with a glucoside (162 Da) loss in molecular weight. Peak 12 showed λ_max_ at 345 nm (band I hypsochromic shift of nearly 21 nm), which was assigned to kaempferol 3-glucoside (Km3g) with the MS data 449.01 ([M–H]^+^) and fragment *m/z* 287.04 ([Y0]^+^). As widely distributed flavonols in many plants (Li et al., 2009; Sarangowa et al., 2014), Qu7g and Km3g are also present in other daylily accessions (Cichewicz and Nair, 2002; Lin et al., 2011; Sun et al., 2018). Peak 4 was characterized as quercetin 3-arabinoside (Qu3ar) because it had fragments at *m/z* 433.08 ([M–H]^–^) and 271 ([Y0]^–^), indicating the loss of an arabinoside (150 Da) (Chen et al., 2010). A similar finding was previously reported in the daylily accession ‘Baihua’ (Sun et al., 2018).

Peak 7 had λ_max_ at 311.18 nm, the MS was 623.09 ([M–H]^–^) and fragment *m/z* 315.02 (losing 309 Da), pointing out to isorhamnetin 3-rutinoside (Is3r) (Deng et al., 2013). The Is3r was previously reported in *H. fulva* (Yanjun et al., 2004) and other plant resources, such as lotus (Deng et al., 2013) and sea buckthorn (Pop et al., 2013). Peaks 10 and 13 were speculated as flavones, luteolin 7-glucoside (Lt7g), and apigenin 7-glucoside (Ap7g), respectively. Lt7g was previously reported in yellow color tree peony (Li et al., 2009) and olive leaves (Lama-Muñoz et al., 2019), while Ap7g has been published in chamomile (Bączek et al., 2019). However, this study is the first to report the two flavones in *Hemerocallis*.

Unfortunately, peaks 1, 2, and 6 were not inferred. Peak 1 had MS 663 ([M–H]^+^), and fragment *m/z* 479 ([Y0]^+^), indicating the tentative compound was isorhamnetin 3-neohesperidoside. However, the isorhamnetin 3-neohesperidoside showed λ_max_ at around 254 nm in previous reports (Deng et al., 2013; Pop et al., 2013), which was not confirmed (λ_max_ = 324.77 nm) in our study. Similarly, peaks 2 and 6 were also confirmed according to λ_max_ spectrum and MS data.

In our study, peaks 3 and 5 showed λ_max_ at 520 nm with more peak area, indicating they were anthocyanins (Mitchell et al., 1998). Two anthocyanin standards, cyanidin 3-rutinoside (Cy3r) and cyanidin 3-glucoside (Cy3g) were used to identify the different compounds by co-elution, and the retention times were 15.40 min and 16.7 min, respectively (Figure 3). Peak 5 showed the same retention time by the two standards, and further MS data indicated it was Cy3r. However, the retention time of peak 3 was earlier than the Cy3g standard with MS 611.09 ([M–H]^+^) and *m/z* 287.1 ([Y0]^+^). Previous studies proved that the polarity of di-glucosides is greater than that of mono-glucosides. Therefore, the elution time is always earlier than mono-glucosides (Yongcheng, 2018), indicating cyanidin 3,5-glucoside (Cy3g5g) was the speculated compound. Cy3r and Cy3g5g are common anthocyanin glycosides and are widely distributed in many plants, such as wild bananas (Kitdamrongsont et al., 2008), tree peony (Zhang et al., 2014), and rose (Zhang et al., 2015).

### Composition and Content of Flavonoids in Different Parts of Floral Organ

**FIGURE 4:**
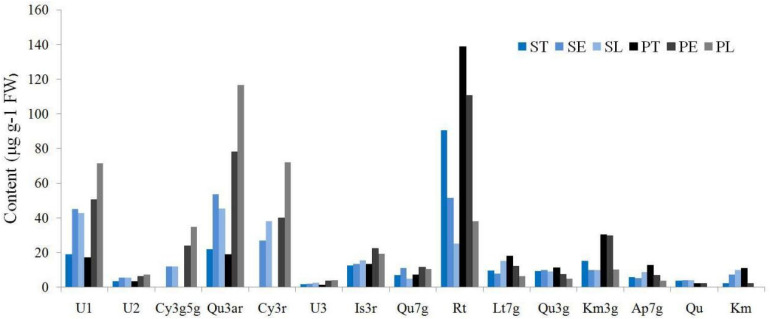
The flavonoids distribution among different floral parts.

The flavonoid composition and contents varied dramatically among the different accessions and parts (Supplementary Table 2), ranging from 0.00 to 321.99 μg g^–1^ FW. Thus, Rt, Qu3ar, and U1 were dominant flavonoids in *Hemerocallis* floral organ, comprising 31.58, 20.66, and 15.61% of relative content, respectively. Our study found that most of the flavonoids we detected were higher in the petal than the sepal (Figure 4). The coefficient of variation (CV) ranged from 25.58 to 186.92% (Table 3). For ST, the flavonol content of Qu had the most considerable CV value (149.57%), followed by U2 (135.21%) and Qu7g (128.16%). For SE, the most significant CV was Qu3g with 163.47%, followed by Cy3r with 143.83% and Km with 133.24%, respectively. For SL, the first three compounds with the most extensive variation range were Rt, Is3r, and Cy3r, with CV values of 141.31, 127.52, and 112.80%, respectively. For PT, the largest variation compound was Km (CV = 158.50%), followed by Qu3g and U2 (CV = 151.81% and 147.89%, respectively). For PE, Qu3g had the largest CV value (178.05%), followed by Cy3r (146.04%), and Is3r (138.68%). For PL, the first three compounds with the largest variation range were Is3r, Cy3g5g, and U2, with CV values of 186.92, 136.45, and 121.40%, respectively.

**TABLE 3:** The difference of flavonoids among different floral parts in the 42 accessions (μg g^–1^ FW).

Peaks	ST				SE				SL				PT				PE				PL			
	Min	Max	Mean	CV (%)	Min	Max	Mean	CV (%)	Min	Max	Mean	CV (%)	Min	Max	Mean	CV (%)	Min	Max	Mean	CV (%)	Min	Max	Mean	CV (%)
U1	1.52	94.20	19.11	95.37	2.04	192.46	45.09	114.41	5.09	167.12	42.80	97.46	0.23	105.82	17.01	116.49	0.29	149.95	50.63	87.40	0.68	229.65	71.51	102.67
U2	0.57	21.38	3.52	135.21	0.99	23.44	5.60	103.15	0.68	26.38	5.62	103.50	0.44	22.65	3.10	147.89	0.20	36.48	6.31	133.23	0.93	38.21	7.22	121.40
Cy3g5g	0.00	0.00	na	na	0.38	55.70	12.14	118.79	0.01	56.57	12.07	112.45	0.00	0.00	na	Na	0.02	124.22	23.71	127.80	0.92	190.53	34.83	136.45
Qu3ar	1.36	115.70	21.88	97.71	1.67	220.66	53.56	103.64	3.61	176.69	45.54	92.52	2.31	108.93	18.80	103.82	2.95	191.76	77.99	72.79	0.73	397.29	116.63	86.82
Cy3r	0.00	0.00	na	na	0.40	126.23	26.99	143.83	0.02	154.52	37.95	112.80	0.00	0.00	na	Na	0.03	230.36	39.91	146.04	1.69	248.90	72.08	104.70
U3	0.74	4.01	1.79	58.04	0.89	5.82	2.17	71.84	0.10	10.23	2.56	106.14	0.18	2.64	1.13	66.43	0.84	17.11	3.46	137.88	0.46	15.69	3.98	116.61
Is3r	1.41	65.25	12.64	105.67	2.69	96.75	13.54	128.54	2.27	112.30	15.64	127.52	1.21	101.10	13.16	144.24	2.28	159.69	22.42	138.68	0.98	167.89	19.35	186.92
Qu7g	0.63	40.88	6.95	128.16	0.01	34.55	11.21	100.03	0.01	14.41	5.01	70.63	0.17	28.92	6.95	124.66	1.15	52.48	11.58	107.81	0.94	40.12	10.59	111.72
Rt	0.67	259.12	90.67	64.56	3.72	254.88	51.50	111.62	1.76	197.96	25.30	141.31	8.29	321.99	138.79	59.45	8.84	309.43	110.76	69.01	1.57	178.96	38.13	117.13
Lt7g	1.45	38.99	9.81	91.10	0.65	47.00	7.98	113.92	1.79	74.65	15.11	106.43	0.96	115.98	17.85	111.02	3.00	40.87	12.00	82.35	1.72	20.25	6.57	80.26
Qu3g	1.31	58.54	9.38	126.82	1.59	83.30	10.10	163.47	1.88	22.97	8.95	85.45	0.71	90.11	11.02	151.81	1.47	72.89	7.26	178.05	1.12	14.08	4.98	74.10
Km3g	2.53	73.86	15.24	91.52	2.14	42.75	9.99	93.57	1.00	24.74	9.83	81.88	3.25	122.13	30.31	107.55	1.42	101.73	29.55	94.21	0.94	41.48	10.38	107.79
Ap7g	1.64	24.29	5.93	90.68	2.10	14.61	5.24	66.04	1.41	29.44	8.78	73.36	1.17	64.31	12.68	100.28	1.25	17.72	6.83	65.21	1.89	7.55	3.91	45.98
Qu	0.84	15.10	3.74	149.57	2.56	6.23	3.99	41.24	1.61	9.72	4.11	82.69	0.32	5.27	2.14	58.29	1.49	3.21	2.13	25.58	0.00	0.00	na	na
Km	2.29	2.29	2.29	na	0.43	14.43	7.43	133.24	1.78	20.81	9.89	80.90	1.58	36.51	10.82	158.50	1.35	2.58	1.97	44.26	0.00	0.00	na	na

*ST, indicates sepal throat; SE, indicates sepal eye; SL, indicates sepal limb; PT, indicates petal throat; PE, indicates petal eye; PL, indicates petal limb; U1, indicates unknown 1; U2, indicates unknown 2; Cy3g5g, indicates cyanidin 3,5-glucoside; Qu3ar, indicates quercetin 3-arobinoside; Cy3r, indicates cyandin 3-rutinoside; U3, indicates unknown 3; Is3r, indicates isorhamnetin-3-rutinoside; Qu7g, indicates quercetin 7-glucoside; Rt, indicates rutin; Lt7g, indicates luteolin 7-glucoside; Qu3g, indicates quercetin 3-glucoside; Km3g, indicates kaempferol 3-glucoside; Ap7g, indicates apigenin 7-glucoside; Qu, indicates quercetin; and Km, indicates kaempferol.*

The different flavonoids showed different distribution patterns in the six floral parts. In the three parts of the sepal, including ST, SE, and SL, three compounds, Rt, Lt7g, and Km3g, could be detected in the ST of all the 42 accessions. Moreover, we did not detect Rt in SE of three accessions, including ‘Nakai’, ‘Green Mystique’, and ‘Little Wine Cup.’ Meanwhile, six accessions detected no Rt in SL, including ‘Canadian Border patrol 2’, ‘Green Mystique’, ‘Bonanza’, ‘Dahuaxuancao’, ‘Frans Hals’, and *H. fulva* var. *kwanso* var. *reasata*. In the three parts of the petal, Rt could be detected in PT of all the 42 accessions, but PE was not detected in ‘Canadian Border Patrol’ and ‘Little Wine Cup.’ Thirteen accessions did not detect Rt in PL.

Hence, Rt was the dominant compound (Figure 4), similar to the findings of previous reports (Sun et al., 2018), and mainly distributed in PT. In addition, we discovered that Rt was more widely distributed in the sepal than the petal, but the average content was higher in the petal than the sepal. Moreover, the Rt contents were higher in the eye (SE and PE) than limb (SL and PL). Qu7g, and Km3g also showed the same distribution pattern. Ap7g nearly had the same distribution pattern, except the limb, that SL (8.78 μg g^–1^ FW) higher than PL (3.91 μg g^–1^ FW).

In this study, the two cyanidin derivatives (Cy3g5g and Cy3r) could not be detected in nightlilies and daylilies with yellow flowers, while only other 20 colored accessions detected cyanidin derivatives. Moreover, the anthocyanin was nearly not detected in the throat (ST and PT) of the 42 accessions (Supplementary Table 2), indicating no anthocyanin contributed to throat pigment coloration. Throat only had three color phenotypes, yellow, yellow-green, and light yellow, similar to our previous investigation (Cui et al., 2019). As shown in Table 3, the average value of Cy3r ranged from 26.99 to 72.08 μg g^–1^ FW, which is higher than Cy3g5g (12.07∼34.83 μg g^–1^ FW). For the Cy3r content, the average SE (26.99 μg g^–1^ FW) was lower than PE (39.91 μg g^–1^ FW). Similarly, the SL also showed a lower average value (37.95 μg g^–1^ FW) than PL (72.08 μg g^–1^ FW). For Cy3g5g content, SE and SL showed lower average values (12.14 μg g^–1^ FW and 12.07 μg g^–1^ FW, respectively) than PE and PL (23.71 μg g^–1^ FW and 34.83 μg g^–1^ FW, respectively). Thus, petals had higher anthocyanin contents than sepals in the 20 colored accessions.

### The Mann–Whitney *U* Test Analysis

The calyx of the *Hemerocallis* flower organ is specialized into a bright colored sepal, which becomes the main ornamental part of the corolla together with petals. However, we revealed that the flavonoid composition and content showed a considerable difference between sepals and petals. Obviously, the flavonoids compositions were different among the various floral parts. This study used the Mann–Whitney *U* non-parametric test via the R language to analyze the difference in ten major compounds among the six floral parts (Table 4). Generally, the diversity in petal compositions was more than the sepal. Eight compounds showed a significant difference (*p* < 0.05) between PT and PL, while only three compounds showed a significant difference (*p* < 0.05) between ST and PT.

**TABLE 4:** The Mann–Whitney *U* test *p*-value of flavonoids between different parts of sepal and petal.

*T*-test	U1	Cy3g5g	Qu3ar	Is3r	Qu7g	Rt	Lt7g	Qu3g	Km3g	Ap7g
ST-SE	0.0418*	1.137e-06***	0.0217*	0.6064	0.9211	5.787e-05***	0.0029**	0.0771	0.0011**	0.3653
ST-SL	0.0022**	1.137e-06***	0.0085**	0.1942	0.5013	7.675e-10***	0.0578	0.0232*	0.0001***	0.5363
SE-SL	0.4285	0.922	0.8615	0.39	0.4418	0.0062**	0.6695	0.6088	0.0519	0.0953
PT-PE	0.0262*	5.033e-07***	0.0177*	0.0045**	0.2571	0.0609	0.0132*	0.2679	0.7848	0.0352*
PT-PL	0.0044**	1.137e-06***	0.0131*	0.7124	0.864	3.846e-11***	1.901e-10***	2.275e-05***	1.868e-07***	3.388e-07***
PE-PL	0.2484	0.9767	0.4188	0.01029*	0.3869	6.152e-08***	7.067e-06***	0.0009***	6.284e-08***	0.0001***
ST-PT	0.0049**	na	0.0273*	0.5329	0.3293	0.0069**	0.0773	0.657	0.2393	0.3997
SE-PE	0.1269	0.5961	0.5142	0.0293*	0.8009	7.812e-05***	0.1111	0.8835	0.0009***	0.8862
SL-PL	0.4389	0.6632	0.9499	0.1862	0.9493	0.5496	0.0003***	0.0396*	0.0491*	9.098e-07***

***Indicates *p* < 0.001, **indicates *p* < 0.01, *indicates *p* < 0.05. ST, indicates sepal throat; SE, indicates sepal eye; SL, indicates sepal limb; PT, indicates petal throat; PE, indicates petal eye; PL, indicates petal limb; U1, indicates unknown 1; Cy3g5g, indicates cyanidin 3,5-glucoside; Qu3ar, indicates quercetin 3-arobinoside; Is3r, indicates isorhamnetin-3-rutinoside; Qu7g, indicates quercetin 7-glucoside; Rt, indicates rutin; Lt7g, indicates luteolin 7-glucoside; Qu3g, indicates quercetin 3-glucoside; Km3g, indicates kaempferol 3-glucoside; and Ap7g, indicates apigenin 7-glucoside.

For sepals, the ST was significantly different from the other two parts. In the Mann–Whitney *U* non-parametric test results between ST and SE, six compounds showed significant differences. For instance, Cy3g5g and Rt showed a significant difference at *p* < 0.001, Lt7g and Km3g at *p* < 0.01, and two compounds (U1 and Qu3ar) at *p* < 0.05. At the same time, ST showed a significant difference with SL in Cy3g5g, Rt, and Km3g at *p* < 0.001, U1 and Qu3ar at *p* < 0.01, and Qu3g at *p* < 0.05. However, only Rt showed a significant difference (*p* < 0.01) between SE and SL.

For petals, the Mann–Whitney *U* non-parametric test results between PT and PE showed five compounds had significant differences, such as one compound (Cy3g5g) at *p* < 0.001 level, one compound (Is3r) at *p* < 0.01 level, and three combinations (Qu3ar, Lt7g, and Ap7g) at *p* < 0.05. Eight compounds showed significant differences between PT and PL, such as Cy3g5g, Rt, Lt7g, Qu3g, Km3g, and Ap7g at *p* < 0.001, U1 at *p* < 0.01, and Qu3ar at *p* < 0.05. The difference between PE and PL was also significant, which was not similar to sepals; five compounds (Rt, Lt7g, Qu3g, Km3g, and Ap7g) were significantly different at *p* < 0.001, and one compound, Is3r, was significantly different at *p* < 0.05.

However, the difference between the same parts in the sepal and petal was not substantial. Only three compounds showed a significant difference between ST and PT, including U1 (*p* < 0.01), Rt (*p* < 0.01), and Qu3ar (*p* < 0.05). The significantly different compounds between SE and PE were Rt and Km3g, *p* < 0.001, and Is3r at *p* < 0.05. Between SL and PL, two compounds (Lt7g and Ap7g) showed significant differences at *p* < 0.001, and two compounds (Qu3g and Km3g) had a considerable difference at *p* < 0.05.

### Hierarchical Cluster Analysis

**FIGURE 5:**
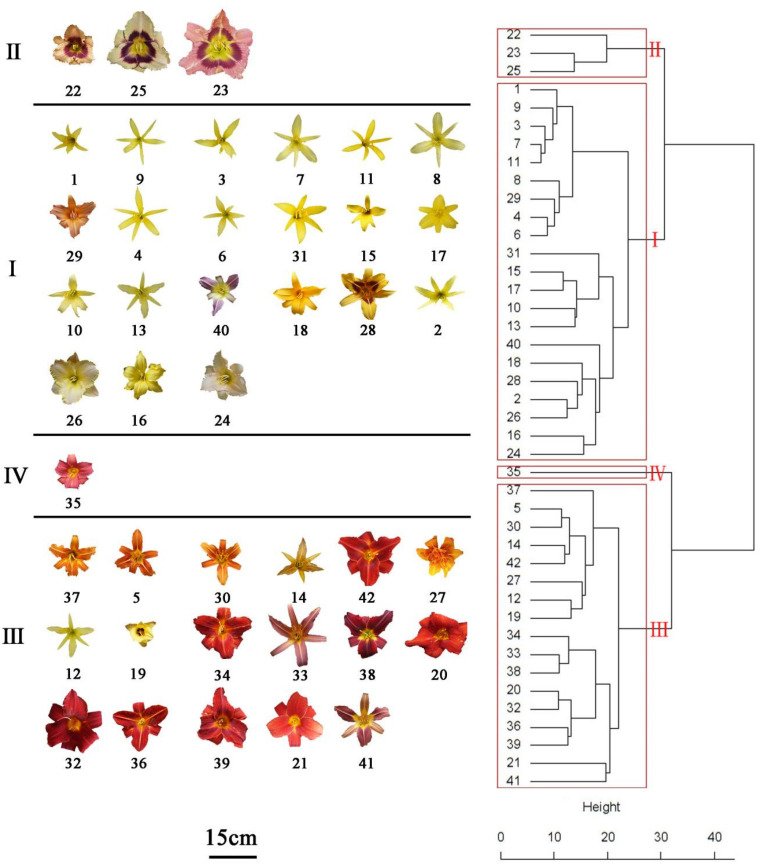
Hierarachical cluster dendritic diagram of 42 *Hemerocallis* accessions.

**FIGURE 6:**
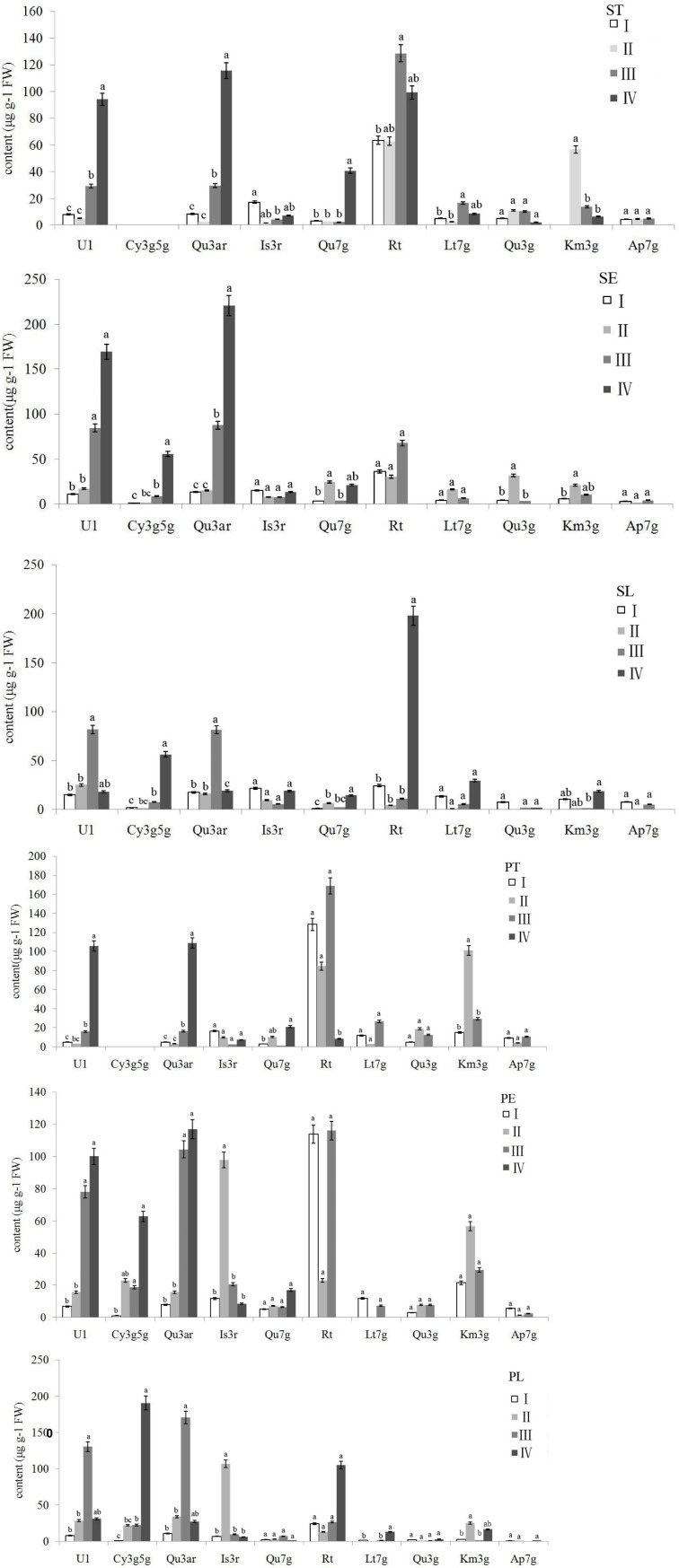
Variations in ten major flavonoids between four clusters. Letters in common are significantly different (*p* < 0.05). ST, sepal throat; SE, sepal eye; SL, sepal limb; PT, petal throat; PE, petal eye; PL, petal limb.

Hierarchical cluster analysis was conducted by Ward’s D method to accurately describe the characteristics among the 42 accessions. Depending on the variations of ten identified flavonoids and anthocyanin components of the accessions, finally, these accessions were grouped into four clusters (Figure 5). Flavonoid compounds, including flavonols, flavones, etc. in leaves were also varied across the amaranth accessions (Sarker and Oba, 2020a,b; Sarker et al., 2020). One-way ANOVA showed a significant difference in the ten significant compounds between four clusters (*p* < 0.05). The variations in ten major compounds between the four groups are shown in Figure 6.

The accessions falling in clusters I and II had lower U1 and Qu3ar relative to other clusters in the six floral parts. The cluster I was composed of 21 accessions, which could be divided into three subgroups. The landraces of nightlilies clustered in the same subgroup, which included two branches. For instance, ‘Datonghuanghua’, ‘Malinhuanghua’, ‘Yanchihuanghua’, and ‘Qiaotouhuanghua’ fell into the same branch; ‘Dongzhuanghuanghua’, ‘Dalihuanghua’, and *H. minor* clustered in another branch of this subgroup. The five compounds of Rt, Lt7g, Qu3g, Km3g, and Ap7g were not detected in PL, but the orange ‘Childrens Festival’ clustered into the same branch, which was contrary to our expectation. Meanwhile, the three accessions, *H. citrine*, *H. thunbergii*, and *H. altissima*, and two landraces, ‘Huohuanghua’, and ‘Qiezi’, clustered in another subgroup. The third subgroup included one night lily accession, one purple daylily, two yellow daylily, and two pink daylily accessions.

The cluster II was composed of three pink accessions, ‘Canadian Border Patrol 2’, ‘Always Afternoon’, and ‘Canadian Border Patrol.’ The pink accessions showed the highest average content of Is3r in PE and PL (97.8 and 107 μg g^–1^ FW, respectively) than other clusters; One-way ANOVA also supported these results (*p* < 0.05). The Km3g content of ST and PT was also significantly higher (*p* < 0.05) than other clusters, with average values 56.78 and 101.25 μg g^–1^ FW, respectively. Although the Km3g content in SE and PE was the highest, the differences were not significant (*p* < 0.05).

A total of 17 accessions, including most orange and red accessions, fell in cluster III; their PE showed higher contents in Qu3ar and Rt than cluster II. The average Qu3ar was 104.4 μg g^–1^ FW, ranging from 11.76 to 191.76 μg g^–1^ FW, while the average Rt was 116 μg g^–1^ FW, varying from 8.84 to 309.43 μg g^–1^ FW. The cluster IV was composed of a single ‘Little wine cup’, and it was the only accession that was not detected in Rt, Lt7g, Qu3g, Km3g, and Ap7g, in SE and PE. Thus, ‘Little wine cup’ could be thought of as a natural mutant to study the loss of Rt metabolism in *Hemerocallis*.

## Conclusion

In conclusion, this is the first report on systematic identification and quantification of flavonols, flavones, and anthocyanins in *Hemerocallis* floral organs. A total of eight flavonols, two flavones, and two anthocyanins were identified. Rutin was the most abundant flavonols in the *Hemerocallis* tepals, followed by quercetin 3-arabinoside, kaempferol 3-glucoside, isorhamnetin 3-rutinoside. Two flavones, luteolin 7-glucoside and apigenin 7-glucoside, were reported for the first time in *Hemerocallis* flowers. The flavonoid composition and content varied dramatically among the different accessions. The different flavonoids showed different distribution patterns in the six floral parts. According to the type and content of flavonoids we detected, the 42 accessions were further divided into 4 groups through hierarchical cluster analysis, and each group had similar flower color phenotypes. These results increased our understanding on the diversity of germplasm resources caused by the differential accumulation and distribution of flavonoid in *Hemerocallis*; and these results is helpful for further studies on the physiol-ecological and molecular mechanisms of flavonoid metabolism pathways in *Hemerocallis*.

## Data Availability Statement

The original contributions presented in the study are included in the article/Supplementary Material, further inquiries can be directed to the corresponding author/s.

## Author Contributions

SL collected the germplasm and designed the project. HC and XX ran the HPLC. FH and JW collected and manage the germplasm. XK and GX advised the manuscript and supported the whole project. All authors contributed to the article and approved the submitted version.

## Conflict of Interest

The authors declare that the research was conducted in the absence of any commercial or financial relationships that could be construed as a potential conflict of interest.

## References

[B1] Bączek K. B. Wiśniewska M. Przybył J. L. Kosakowska O. Wêglarz Z. (2019). Arbuscular mycorrhizal fungi in chamomile (*Matricaria recutita* L.) organic cultivation. *Ind. Crops Prod.* 140:111562.

[B2] Chen H. Huang Y. Qu J. (2012). Dertermination of rutin, quercetin and kaempferol in alabastrum *Hemerocallis fulva* from different origin in Gansu province. *J. Pharm. Anal.* 09 1574–1577. 10.16155/j.0254-1793.2012.09.013

[B3] Chen H. Zhou X. Cao G. Gan X. (2010). Determination of quercetin 3-O-α-L-arabinopyranoside in *Periploca forrestii* by RP-HPLC. *J. Chin. Mater. Med.* 35 1284–1286. 10.4268/cjcmm20101014 20707198

[B4] Cichewicz R. H. Nair M. G. (2002). Isolation and characterization of stelladerol, a new antioxidant naphthalene glycoside, and other antioxidant glycosides from edible daylily (*Hemerocallis*) flowers. *J. Agric. Food Chem.* 50 87–91. 10.1021/jf010914k 11754548

[B5] Cichewicz R. H. Zhang Y. Seeram N. P. Nair M. G. (2004). Inhibition of human tumor cell proliferation by novel anthraquinones from daylilies. *Life Sci.* 74 1791–1799. 10.1016/j.lfs.2003.08.034 14741736

[B6] Cos P. De Bruyne T. Hermans N. Apers S. Berghe D. V. Vlietinck A. J. (2004). Proanthocyanidins in health care: current and new trends. *Curr. Med. Chem.* 11 1345–1359. 10.2174/0929867043365288 15134524

[B7] Cui H. Zhang Y. Shi X. Gong F. Xiong X. Kang X. (2019). The numerical classification and grading standards of daylily (*Hemerocallis*) flower color. *PLoS One* 14:e216460. 10.1371/journal.pone.0216460 31170177 PMC6553707

[B8] Deng F. Yin H. Li J. Hong Y. (2003). On latest application and countermeasure for industrialization exploitation of daylily flower. *J. Hum. Agric. Univ.* 29 529–532. 10.13331/j.cnki.jhau.2003.06.019

[B9] Deng J. Chen S. Yin X. Wang K. Liu Y. Li S. (2013). Systematic qualitative and quantitative assessment of anthocyanins, flavones and flavonols in the petals of 108 lotus (*Nelumbo nucifera*) cultivars. *Food Chem.* 139 307–312. 10.1016/j.foodchem.2013.02.010 23561110

[B10] Fernandes L. Casal S. Pereira J. A. Saraiva J. A. Ramalhosa E. (2017). Edible flowers: A review of the nutritional, antioxidant, antimicrobial properties and effects on human health. *J. Food Compost. Anal.* 60 38–50. 10.1016/j.jfca.2017.03.017

[B11] Fu M. Mao L. (2006). A review of the research on the health efficacy and chemical constituents of daylily (*Hemerocallis fulva*). *Food Ferment. Ind.* 10 108–112.

[B12] Hsu Y. Tsai C. Chen W. Ho Y. Lu F. (2011). Determination of lutein and zeaxanthin and antioxidant capacity of supercritical carbon dioxide extract from daylily (*Hemerocallis disticha*). *Food Chem.* 129 1813–1818. 10.1016/j.foodchem.2011.05.116

[B13] Kao F. J. Chiang W. D. Liu H. M. (2015). Inhibitory effect of daylily buds at various stages of maturity on nitric oxide production and the involved phenolic compounds. *LWT* 61 130–137. 10.1016/j.lwt.2014.11.023

[B14] Kitdamrongsont K. Pothavorn P. Swangpol S. Wongniam S. Atawongsa K. Svasti J. (2008). Anthocyanin Composition of Wild Bananas in Thailand. *J. Agric. Food Chem.* 56 10853–10857. 10.1021/jf8018529 18959407

[B15] Lama-Muñoz A. Contreras M. D. M. Espínola F. Moya M. Romero I. Castro E. (2019). Optimization of Oleuropein and Luteolin-7-O-Glucoside Extraction from Olive Leaves by Ultrasound-Assisted Technology. *Energies* 12:2486. 10.3390/en12132486

[B16] Li C. Du H. Wang L. Shu Q. Zheng Y. Xu Y. (2009). Flavonoid Composition and Antioxidant Activity of Tree Peony (*Paeonia* Section *Moutan*) Yellow Flowers. *J. Agric. Food Chem.* 57 8496–8503. 10.1021/jf902103b 19711909

[B17] Li S. Shi Q. Hou F. Chen Z. Ji F. Dang H. (2016). Diversity of the main ornamental traits for *Hemerocallis* spp. accessions. *J. Shanxi Agric. Univ.* 09 619–627. 10.13842/j.cnki.issn1671-8151.2016.09.003

[B18] Lin Y. Lu C. Huang Y. Chen H. (2011). Antioxidative Caffeoylquinic Acids and Flavonoids from *Hemerocallis fulva* Flowers. *J. Agric. Food Chem.* 59 8789–8795. 10.1021/jf201166b 21761841

[B19] Manach C. Scalbert A. Morand C. Remesy C. Jimenez L. (2004). Polyphenols: food sources and bioavailability. *Am. J. Clin. Nutr.* 79 727–747. 10.1093/ajcn/79.5.727 15113710

[B20] Markham K. R. (1989). “Flavones, flavonols and their glycosides,” in *Methods Plant Biochemistry*, ed. Harborne J. B. (Cambridge: Academic Press), 197–235.

[B21] Mitchell K. Markham K. R. Boase M. R. (1998). Pigment chemistry and colour of Pelargonium flowers. *Phytochemistry* 47 355–361. 10.1016/s0031-9422(97)00595-5

[B22] Pop R. M. Socaciu C. Pintea A. Buzoianu A. D. Sanders M. G. (2013). UHPLC/PDA-ESI/MS Analysis of the Main Berry and Leaf Flavonol Glycosides from Different Carpathian *Hippophaë rhamnoides* L. Varieties. *Phytochem. Anal.* 24 484–492. 10.1002/pca.2460 24038430

[B23] Rodriguez-Enriquez M. J. Grant-Downton R. T. (2013). A new day dawning: *Hemerocallis* (daylily) as a future model organism. *AoB Plants* 5:s55. 10.1093/aobpla/pls055 23440613 PMC3580041

[B24] Sarangowa O. Kanazawa T. Nishizawa M. Myoda T. Bai C. Yamagishi T. (2014). Flavonol glycosides in the petal of Rosa species as chemotaxonomic markers. *Phytochemistry* 107 61–68. 10.1016/j.phytochem.2014.08.013 25220498

[B25] Sarker U. Oba S. (2018). Drought stress enhances nutritional and bioactive compounds, phenolic acids and antioxidant capacity of *Amaranthus* leafy vegetable. *BMC Plant Biol.* 18:258. 10.1186/s12870-018-1484-1 30367616 PMC6203965

[B26] Sarker U. Oba S. (2019). Antioxidant constituents of three selected red and green color *Amaranthus* leafy vegetable. *Sci. Rep.* 9:18233. 10.1007/s11295-011-0447-6 PMC6890792 31796754

[B27] Sarker U. Oba S. (2020c). Polyphenol and flavonoid profiles and radical scavenging activity in leafy vegetable *Amaranthus gangeticus*. *BMC Plant Biol.* 20:499. 10.1186/s12870-020-02700-0 33138787 PMC7607633

[B28] Sarker U. Oba S. (2020d). The Response of Salinity Stress-Induced A. tricolor to Growth, Anatomy, Physiology, Non-Enzymatic and Enzymatic Antioxidants. *Front. Plant Sci.* 11:559876. 10.3389/fpls.2020.559876 33178233 PMC7596248

[B29] Sarker U. Oba S. (2020b). Phenolic profiles and antioxidant activities in selected drought-tolerant leafy vegetable amaranth. *Sci. Rep.* 10:18287. 10.1038/s41598-020-71727-y 33106544 PMC7589480

[B30] Sarker U. Oba S. (2020a). Nutraceuticals, phytochemicals, and radical quenching ability of selected drought-tolerant advance lines of vegetable amaranth. *BMC Plant Biol.* 20:564. 10.1186/s12870-020-02780-y 33317465 PMC7737358

[B31] Sarker U. Hossain M. N. Iqbal M. A. Oba S. (2020). Bioactive Components and Radical Scavenging Activity in Selected Advance Lines of Salt-Tolerant Vegetable Amaranth. *Front. Nutr.* 7:587257. 10.3389/fnut.2020.587257 33330589 PMC7734134

[B32] Singh R. Wu B. Tang L. Liu Z. Hu M. (2010). Identification of the Position of Mono-O -glucuronide of Flavones and Flavonols by Analyzing Shift in Online UV Spectrum (λ_max_) Generated from an Online Diode Array Detector. *J. Agric. Food Chem.* 58 9384–9395. 10.1021/jf904561e 20687611 PMC3404750

[B33] Sun J. Liu W. Zhang M. Geng P. Shan Y. Li G. (2018). The analysis of phenolic compounds in daylily using UHPLC-HRMS_*n*_ and evaluation of drying processing method by fingerprinting and metabolomic approaches. *J. Food Process Pres.* 42:e13325. 10.1111/jfpp.13325

[B34] Tai C. Chen B. (2000). Analysis and stability of carotenoids in the flowers of daylily (*Hemerocallis disticha*) as affected by various treatments. *J. Agric. Food Chem.* 48 5962–5968. 10.1021/jf000956t 11312769

[B35] Wang A. Li R. Ren L. Gao X. Zhang Y. Ma Z. (2018). A comparative metabolomics study of flavonoids in sweet potato with different flesh colors (Ipomoea batatas (L.) Lam). *Food Chem.* 260 124–134. 10.1016/j.foodchem.2018.03.125 29699652

[B36] Wang X. Gao Y. (2014). *Hemerocallis*, 1st Edn. Beijing: China Forestry Press.

[B37] Wiseman S. A. Mathot J. N. de Fouw N. J. Tijburg L. B. (1996). Dietary non-tocopherol antioxidants present in extra virgin olive oil increase the resistance of low density lipoproteins to oxidation in rabbits. *Atherosclerosis* 120 15–23. 10.1016/0021-9150(95)05656-4 8645356

[B38] Yanjun Z. Chichewicz R. H. Nair M. G. (2004). Lipid peroxidation inhibitory compounds from daylily (*Hemerocallis fulva*) leaves. *Life Sci.* 75 753–763. 10.1016/j.lfs.2004.07.001 15172183

[B39] Yongcheng N. (2018). *Structural Identification of Organic Compounds with Spectroscopic Techniques*, 4th Edn. Beijing: Science Press.

[B40] Zhang C. Wang W. Wang Y. Gao S. Du D. Fu J. (2014). Anthocyanin biosynthesis and accumulation in developing flowers of tree peony (*Paeonia suffruticosa*) ‘Luoyang Hong’. *Postharvest. Biol. Technol.* 97 11–22. 10.1016/j.postharvbio.2014.05.019

[B41] Zhang L. Xu Z. Tang T. Zhang H. Zhao L. (2015). Analysis of anthocyanins related compounds and their biosynthesis pathways in Rosa rugosa ‘Zi Zhi’ at blooming stages. *Sci. Agri. Sin.* 13 2600–2611. 10.3864/j.issn.0578-1752.2015.13.012

[B42] Zloch Z. (1996). The role of dietary plant polyphenols in health maintenance. *Cas Lek Cesk* 135 84–88. 8625375

